# Randomized controlled trial protocol to evaluate the effect of an educational intervention using information, motivation and behavioral skills model on sexual satisfaction of new couples in Iran

**DOI:** 10.1186/s12978-019-0821-7

**Published:** 2019-11-15

**Authors:** Babak Moeini, Effat Merghati Khoei, Majid Barati, Alireza Soltanian, Manoj Sharma, Reza Khadivi, Ali Ghaleiha, Vinayak K. Nahar, Fahimeh Bagherikholenjani

**Affiliations:** 1 Social Determinants of Health Research Center and Department of Public Health, School of Public Health, Hamadan University of Medical Sciences, Hamadan, Iran; 2 The Iranian National Center for Addiction Studies (INCAS), Tehran University of Medical Sciences, Tehran, Iran; 3 Director of the Family-Sexual Health Division in the Brain and Spinal Cord Injury Research Center (BASIR), Neuroscience Institution, Tehran University of Medical Sciences, Tehran, Iran; 4 Modeling of Non communicable Diseases Research Center and Department of Biostatistics, School of Public Health, Hamadan University of Medical Sciences, Hamadan, Iran; 5 Department of Behavioral and Environmental Health, School of Public Health, Jackson State University, Jackson, MS USA; 6 Health for All, Inc., Omaha, NE USA; 7 Department of Community Medicine, Medical School, Isfahan University of Medical Sciences, Isfahan, Iran; 8 Department of Psychiatry, School of Medicine, Hamadan University of Medical Sciences, Hamadan, Iran; 9 Department of Dermatology, School of Medicine, The University of Mississippi Medical Center, Jackson, MS USA; 10 Department of Preventive Medicine, School of Medicine/John D. Bower School of Population Health, The University of Mississippi Medical Center, Jackson, MS USA; 11 School of Public Health, Hamadan University of Medical Science, Hamadan, Iran

**Keywords:** Protocol study, Couple, Sexual satisfaction, Sex education, Information motivation behavioral skill model, Iran, estudio de protocolo, pareja, satisfacción sexual, educación sexual, modelo de habilidad de comportamiento de motivación de información, Irán, étude de protocole, couple, satisfaction sexuelle, éducation sexuelle, modèle de compétences comportementales de la motivation par l’information, Iran

## Abstract

**Background:**

Sexual satisfaction is considered as one of the key factors in assessing a person’s quality of life and the quality and continuity of marital relationships. According to the results of reports in Iran, many couples are dissatisfied with their sexual lives. Sexuality education is one of the important strategies to prevent early sexual problems and improve sexual satisfaction. The aim of this randomized controlled trial is to compare the efficacy of sexual and marital enrichment package using information, motivation and behavioral skills model on sexual satisfaction of new couples in Iran to routine sexual care program that provided at governmental health centers.

**Methods:**

This is a randomized, controlled, superiority trial with two parallel groups. One hundred new couples (*n* = 200) will be recruited and randomized with simple randomization method and a 1:1 allocation. Recruitment will be from governmental health centers and calling on social networks. Couples will be randomized to intervention which will receive Sexual and Marital Enrichment package and control group (routine care at health centers). Couples will be followed up for 4 months. Then primary outcomes (mean score of couples’ sexual information, motivation and behavior skills) and secondary outcome (mean score of couples’ sexual satisfaction) of study will be measured through the online questionnaire.

**Discussion:**

This trial will be examined the impact of the sexual and marital skills training package tailored to the values and norms governing the sexual life of Iranian couples on their sexual satisfaction. If the trial is effective, its results will be presented to policy makers for implementation at national level.

**Trial registration:**

(Iranian Registry of Clinical Trials (IRCT) number): IRCT20181211041926N1. Date of registration: March 2, 2019.

## Resumen

**Antecedentes:**

La satisfacción sexual se considera uno de los factores clave para evaluar la calidad de vida de una persona y la calidad y continuidad de las relaciones matrimoniales. Según los resultados de los informes en Irán, muchas parejas no están satisfechas con su vida sexual. La educación sexual es una de las estrategias importantes para prevenir problemas sexuales tempranos y mejorar la satisfacción sexual. El objetivo de este ensayo controlado aleatorio es comparar la eficacia del paquete de enriquecimiento sexual y matrimonial utilizando información, motivación y modelo de habilidades conductuales sobre la satisfacción sexual de las nuevas parejas en Irán con el programa de atención sexual de rutina que se brinda en los centros de salud gubernamentales.

**Métodos:**

Este es un ensayo aleatorizado, controlado, de superioridad con dos grupos paralelos. 100 nuevas parejas (*n* = 200) serán reclutadas y aleatorizadas con un método de aleatorización simple y una asignación 1: 1. El reclutamiento se realizará en centros de salud gubernamentales y en las redes sociales. Las parejas serán aleatorizadas para recibir una intervención que recibirá un paquete de enriquecimiento sexual y marital y un grupo de control (atención de rutina en los centros de salud). Las parejas serán seguidas durante 4 meses. Luego, los resultados primarios (puntaje promedio de información sexual de las parejas, motivación y habilidades de comportamiento) y el resultado secundario (puntaje promedio de satisfacción sexual de las parejas) del estudio se medirán a través del cuestionario en línea.

**Discusión:**

Este ensayo examinará el impacto del paquete de capacitación en habilidades sexuales y matrimoniales adaptado a los valores y normas que rigen la vida sexual de las parejas iraníes en su satisfacción sexual. Si el ensayo es efectivo, sus resultados se presentarán a los encargados de formular políticas para su implementación a nivel nacional.

## Abstrait

**Contexte:**

La satisfaction sexuelle est. considérée comme l’un des facteurs clés dans l’évaluation de la qualité de vie, et de la continuité des relations conjugales. Selon les résultats de rapports en Iran, de nombreux couples sont insatisfaits de leur vie sexuelle. L’éducation sexuelle est. l’une des stratégies importantes pour prévenir les problèmes sexuels précoces et améliorer la satisfaction sexuelle. L’objectif de cet essai contrôlé randomisé est. de comparer l’efficacité de l’enrichissement sexuel et conjugal à l’aide du modèle d’information, de motivation et de compétences comportementales sur la satisfaction sexuelle des nouveaux couples en Iran avec le programme de soins de routine dispensé dans les centres de santé gouvernementaux.

**Les méthodes:**

Il s’agit d’un essai de supériorité randomisé et contrôlé avec deux groupes parallèles. 100 nouveaux couples (*n* = 200) seront recrutés et randomisés avec une méthode de randomisation simple et une allocation 1: 1. Le recrutement se fera dans les centres de santé gouvernementaux et fera appel aux réseaux sociaux. Les couples seront randomisés pour une intervention qui recevra un paquet d’enrichissement sexuel et matrimonial et un groupe témoin (soins de routine dans les centres de santé). Les couples seront suivis pendant 4 mois. Ensuite, les résultats primaires (score moyen d’informations sexuelles des couples, motivation et compétences comportementales) et les résultats secondaires (score moyen de satisfaction sexuelle des couples) de l’étude seront mesurés à l’aide du questionnaire en ligne.

**Discussion:**

Ce procès examinera l’impact du programme de formation en compétences sexuelles et matrimoniales adapté aux valeurs et aux normes régissant la vie sexuelle des couples iraniens en ce qui concerne leur satisfaction sexuelle. Si l’essai est. efficace, ses résultats seront présentés aux décideurs pour qu’ils soient mis en œuvre au niveau national.

**Inscription à l’essai:**

(Numéro du registre iranien des essais cliniques (IRCT)): IRCT20181211041926N1.

## Plain English summary

Sexual satisfaction is a component of human sexuality that is considered as the ultimate stage of one’s sexual response. According to the results of reports in Iran, many couples are dissatisfied with their sexual lives. Sexuality education is one of the important strategies to prevent early sexual problems and improve sexual satisfaction. The present study is a randomized controlled trial (RCT) that aims to compare the efficacy of sexual and marital skills training package using information, motivation and behavioral skills model on sexual satisfaction of new couples in Iran to routine sexual care program that provided at governmental health centers. The training package includes the 18-h Marital and Sexual Skills Training Workshop, the presentation of the “My Soul Partner” book and sending short messages. Couples in control group receive only the reproductive health care provided at the urban health centers. We expect that sexual and marital skills training package that tailored to the values and norms governing the sexual life of Iranian couples can improve the level of sexual information, motivation and behavioral skills of the couples in intervention group compared to the control group 4 months after the end of intervention. Consequently, we expect to show that training package improves the sexual satisfaction of couples in intervention group compared to the control group.

## Background

Sexual satisfaction in marital relationships is generally considered as one of the key factors in assessing a person’s quality of life and the quality and continuity of marital relationships in particular [1], which is closely associated to the structures related to the quality of marital life, such as communication between couples [2] and marital satisfaction [3]. Sexual satisfaction is a component of human sexuality that is considered as the ultimate stage of one’s sexual response [4]. The concept of sexual satisfaction is divided into two types of satisfaction with sexual activity and emotional satisfaction [5]. In fact, sexual satisfaction is a delightful feeling of individual behavior and interpersonal interactions that is defined as analysis and judgment of one’s own sexual behavior [6].

Sexual satisfaction is correlated with physical and mental health, general happiness, successful social interactions and professional achievements [7]. Sexual satisfaction is also associated with quality of relationship, such as supporting relationships, emotional empathy, physical attractiveness, love, commitment and stability [8]. So that excessive stress caused by sexual dissatisfaction can lead to physical and mental disorders and reduce individual abilities and creativity [9] Hulbert and colleagues state that dissatisfaction with sexual function is closely related not only to divorce but also to some social problems, such as crimes, sexual assault, and mental illnesses. In this regard, Bentovim, quoted from Masters and Johnson, considers the causes of failure of 50% of marriages related to sexual dissatisfaction [10]. According to the results of reports in Iran, many couples are dissatisfied with their sexual lives. Almost 50 to 60% of divorces and 40% of betrayals and covert communication are related to sexual dissatisfaction [9, 11, 12]. One of the problems in the traditional Iranian society is lack of sufficient information on sex and having erroneous attitudes and beliefs about this issue in families, and in particular among new couples. For example, one of the false attitudes that are more common among women is that taking about sex is a sin. This type of attitude prevents a proper response to the legitimate needs of their spouses; so that they not only do not consent to their sexual relationship but also not allow their spouse to enjoy their natural and emotional desires [13]. These shortcomings are often the result of misunderstandings related to sexuality and social and cultural barriers for sex education and lack or low level of quantity and quality of sexual health services [14]. Regarding these issues, one of the important strategies is sexuality education to prevent early sexual problems and promote sexual health. The results of few studies conducted in Iran [15–17] show that sexuality education is effective in improving sexual satisfaction and ultimately marital satisfaction. Given that the sexual education encompasses three domains included of the cognitive (information and knowledge), emotional (emotions, values and attitudes) and behavioral (communication and sexual skills) domains [18]; the Information, Motivation and Behavioral skills model will be used as a theoretical framework in this interventional study.

### Theoretical framework: information–motivation–behavioral skills model

The IMB model conceptualizes psychological determinants of the performance of behaviors that have the valence to destroy, or to modify health status. This model is a useful framework for our study that specifically addresses the sexual information, motivation and behavioral skills needs of young Iranian couples to improve their sexual health. According to the IMB model, information is a fundamental determinant of sexual health behavior performance that can be easily enacted by an individual in his or her social ecology [19]. Information includes specific facts about sexual health promotion, also relative heuristics (plain rules that permit cognitively effortless and automatic– but often incorrect – decisions about whether or not involved in sexual behavior)**.** Third component of information included relatively elaborate implicit theories (complex beliefs that are often incorrect and require cognitive attempt to process in deciding about sexual behaviors). The next structure of IMB model which effects on sexual health promoting behaviors is motivation that include personal motivation (attitudes toward personal performance of sexual health promoting behaviors) and social motivation (social support for enactment of sexual health promotion behaviors). Behavioral skills are an additional principal determinant of IMB models that focuses on an individual’s visual abilities and his or her sensation of self-efficacy [20].

### Objectives and hypothesis

The aim of this randomized controlled trial is to compare the efficacy of sexual and marital skills training package using information, motivation and behavioral skills model on sexual satisfaction of new couples in Iran to routine sexual care program that provided at governmental health centers. It is hypothesized that sexual and marital skills training package will increase sexual information, sexual motivation and sexual behavioral skills of participating couples which will improve sexual satisfaction and ultimately their sexual health compared to control group couples.

### Trial design

This trial is designed as a randomized, controlled, superiority trial with two parallel groups. Randomization will be performed with a 1:1 allocation.

### Study overview

**Fig. 1:**
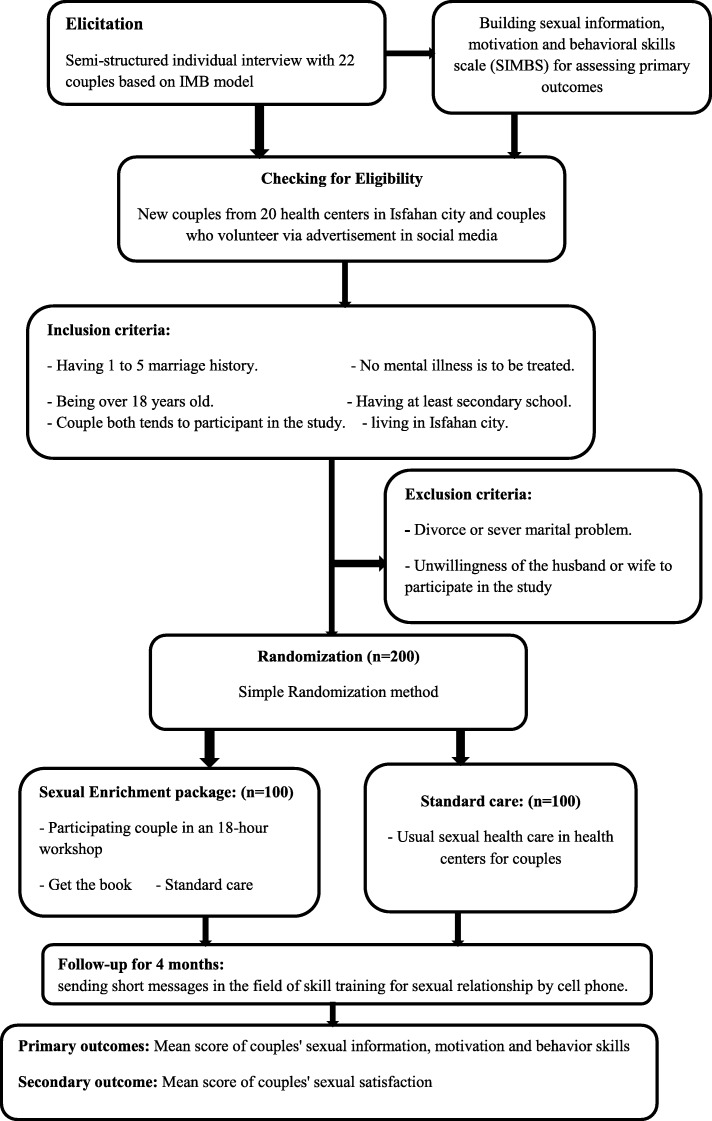
Flow diagram of study

In sexual health interventions using the IMB model, the first step is to focus on elicitation research that aims to empirically identify information deficits, motivational barriers, behavioral skills limitations and assets and general sexual behavior faced in a representative subsample of a target population [20]. For this purpose, semi-structured individual interviews were conducted based on IMB model with 22 heterosexual couples (22 males and 22 females). Based on extracted items from contributing couple interviews, designed the sexual information, motivation and behavioral skills scale (SIMBS) for Iranian couples, and its psychometric properties were confirmed in a sample of 200 couples (400 persons) [21]. SIMBS will be used to assess the initial outcomes of the intervention. The second step involves designing and presenting empirically targeted, conceptually based, population-specific interventions constructed on the basis of elicitation research findings. Therefore, at this stage, a two arms randomized controlled trial will be designed and implemented according to findings from the previous step to address empirically identified deficits in sexual information, motivation and behavioral skills related to sexual satisfaction of couples. The third step is the evaluation of the effect of these interventions after four months follow up to determine whether this interventions have had significant effects on the information, motivation, and behavioral skills as determinants of couples’ sexual satisfaction (Fig. 1- Flow Diagram).

## Methods

### Study setting

This study will be conducted in Isfahan city, the capital of Isfahan province in center of Iran.

According to the statistics center of Iran in 2017, the population of Isfahan city was 1,961,260 people. The number of marriages in Isfahan city was 15,409 in 2017 and the number divorces in the same year was 5067. Approximately 64% of divorces occur in the first five years of life [22]. The setting of the study will be at the urban health center in Isfahan city. In urban areas, health centers are the first level of contact between families and governmental health system. Their staff consists of a qualified physician and a team of up to 10 health workers that provide primary, secondary, and tertiary health services to all age groups from birth to death. In Isfahan city, there are 81 health centers in different parts of the city offering numerous health services to clients. This health centers is managed by district health centers, under the supervision of Isfahan University of Medical Sciences.

### Participant selection

#### Inclusion and exclusion criteria

All couples who are referred to health centers of Isfahan city and are willing to participate in the study or volunteer couples that declare their interest through virtual social network to the researcher will be potential participants in the trial if they meet the inclusion criteria. Couples who have a 1 to 5 years marriage history, are over 18 years old, have at least a degree in secondary schools, living in Isfahan city, do not have any severe mental illness (according to history taking by the main researcher (FB)), both couples express agreement to participate in the study and have a cell phone will be eligible to enter the study. The first criterion is the definition of new or recently married couples [23]. Couples who meet the inclusion criteria but have been divorced or have severe marital problems or husband or wife is unwilling to participate in the study will be excluded from the trial.

#### Recruitment

Informing couples will be done through an invitation entitled “Sexual Relationships Enrichment of New Couples” as a poster that will be installed on the bulletin boards at each of the health center. At the same time, the invitation will be published online on the virtual social network. In health centers, one of the health providers will also be trained about the goals of study and the sampling method. The couples who are eligible to study will be identified by trained health providers and the objectives and conditions of study will be explained to them. Couples can also contact to research team through social network that will be screened for eligibility. Additional information will be given to eligible couples who agree to participate in the trial and their contact numbers will be recorded for inviting in the meetings. All eligible couples who are willing to participate in the study will sign a written consent in Persian language before entering to the trial. The participation will be voluntary and they could withdraw from the study at any time.

#### Randomization

In this study, a simple randomization method will be used. The unit of randomization will be a couple. Firstly, eligible couples will be assigned randomly to either the intervention or the control group according to the Random Number Table, and then the randomization will continue until the balance is established in terms of the size of the groups. In order to ensure allocation concealment, the central randomization method will be used so that a random sequence was given to the original researcher at one of the centers and the samplings of couples in other centers are done simultaneously. Other colleagues at other centers will contact the researcher through the telephone and ask questions about the random allocation of the couple to the special group.

#### Intervention

The intervention that will be presented and evaluated in this trial will be a training package for the enrichment of couples’ sexual interactions, which will enhance the sexual satisfaction of couples (Table 1). This intervention will be compared with the usual care provided at the urban health centers.

**Table 1:** Headings and Learning objectives of the enrichment of couples’ sexual interactions training package

Heading	Learning objective
The importance of skills teaching in marital relationships	Understanding the importance of sex education in the formation of appropriate sexual life Understanding the importance of proper sexual interactions in marital life
Effective communication of couples	Knowing the concept of communication Understanding the growing steps of marriage Understanding the effective communication factors with the spouse Recognizing effective cases in the strength or destruction of communications Earn the ability to talk to spouse about sex
Establish effective communication	The Impact of communication on effective Interpersonal Interactions Communicating to express feelings and ideas, Individual responsibility Good listener features Effective communication skills practice The influence of culture in communication patterns
Anatomy and function of sexual and reproductive organs	Knowing of sexually-reproductive organs of men and women Familiarity with the performance of sexually-reproductive organs of men and women Understanding male and female sexual responses
Vocabulary and sexual response patterns	Understanding sexual vocabulary Understanding the stages of sexual intercourse between husband and wife comprehending the cycle of sexual behaviors in men and women
Skills in sexual relationships	Familiar with the factors affecting good sexual interactions perceiving the basics of sexual life Determine the sexual lifestyle of couples by themselves Understanding the stages of sexual relationship Talking with spouse about the priorities of sexual life, tendencies, fantasies, types of touches.
Sexual health	Understanding the Importance of Sexual Diseases Understanding the factors that cause sexually transmitted diseases Understanding transferring ways of sexually transmitted diseases Familiarity with the symptoms of sexually transmitted diseases Identifying ways to prevent sexually transmitted diseases
Sexual relationships during pregnancy	Recognizing communication problems during pregnancy Understanding physiology of pregnancy Familiarity with how to have sex during pregnancy Learn important tips on sexual relationships in different months of pregnancy Familiarity with different modes of doing sex during pregnancy Recognition of sexual behaviors in the postpartum period
Sexual problems	Identifying common causes of sexual problems Understanding couples from their sexual life inhibitors Identifying couples’ sexual problems by themselves Understanding sexual dysfunctions in men and women Recognizing the effective factors in creating sexual disorders in men and women Action to treat sexual dysfunction

#### Description of intervention

The training package of enrichment of couples’ sexual interactions were designed based on the content of individual interviews with 22 couples in the qualitative phase and the results of the cross-sectional phase of study (*n* = 400) and according to the IMB model. This package includes the 18-h Marital and Sexual Skills Training Workshop, the presentation of the “My Soul Partner” book which is given to each couple for free and sending short messages.

The “My Soul Partner” book is written by second author who is **sexo**logist. The aim of this book is skill training of intimate and sexual relationships of couples in the form of religious and cultural values and norms of the Iranian community that empower couples to have a healthy and intimate marital life. The book forms the educational content of the workshop, which includes the topics of the effective communications of spouses, the anatomy and performance of reproductive organs, the vocabulary and patterns of human sexual response, the sexual communication skills, sexual relationships during pregnancy, sexual health and sexual problems. The content will be presented using collaborative approaches such as, the group discussion, role playing and question and answer in the workshop, and the couples will solve the exercises and self-tests in the book and discuss the results. The 18-h workshop will be planned in the form of six 3-h workshop in 6 consecutive weeks that will be considered rest and catering among meeting sessions for participants. The location of workshops will be at one of the health centers in the center of the city. It is convenient to move all participants from different parts of the city at this central location. The time of holding the workshop will be determined by the participants and will be tried in a way that does not interfere with the working hours of the participants. Teaching in workshops will be handled by two people from the research team.

#### Description of the control

Couples in control group will not receive an intervention after completing the baseline questionnaire, and receive only the reproductive health care provided at the urban health centers. The services provided by the family and community health unit in urban health centers for couples include family planning, counseling and treatment for STDs and HIV/AIDS, premarital training, counseling and screening for reproductive system cancers including breast and cervical cancer. But there is no service in the field of sexual health. Couples in two groups receive routine services.

### Outcome measures

#### Primary outcomes

The primary outcomes of study include the facts that couples will learn about the correct sexual interactions, the identification of the right and wrong sexual beliefs, the attitude towards sexual relations, understanding subjective and social norms in the field of sexual health promotion behaviors, objective skills for the correct sexual behavior and self-efficacy in using these skills in sexual life situations and couples sexual behaviors. Mean score of couples’ sexual information, motivation and behavioral skills will be assessed 4 months after the end of intervention.

#### Secondary outcomes

Mean score of couples’ sexual satisfaction at 4 months after the end of the intervention.

#### Outcome assessment

The outcomes will be measured through the online individual, close ended questionnaire, which is designed on Google Form (https://docs.google.com/forms) and its link will be sent to mobile phone’ participants via accessible social networks (Telegram, WhatsApp messenger, Instagram, IMO and LinkedIn). Given the confidentiality of the questions, this method has been chosen to enhance the privacy of participants and feel more comfortable in answering the questions. Only the main investigator will be able to view the responses. The questionnaires will be administered at the baseline (before assigning couples to the study arms) and 4 months after the end of the intervention. To evaluate the primary outcomes, the SIMBS questionnaire will be used which was designed by the research team based on individual interviews with couples and according to the IMB model and its psychometric properties were confirmed in 200 couples (*n* = 400). To evaluate the secondary outcome, Larson’s Sexual Satisfaction Questionnaire [24] will be used, whose validity and reliability has been confirmed in Iranian couples [8]. The main investigator will contact the couples at the baseline and four months after the intervention and explain how they will fill the questionnaire. This description will be sent in writing along with the questionnaire and instructions for completing the questionnaire. Also, a phone number will be given to couple to contact the research team if they have any questions. Then the questionnaire link will be sent to them. All participants will send their responses via mobile phone. The proper security measures will be taken to assured and arcane transfer data to a secure database.

### Duration of the study

The duration of the intervention from entry to study until the evaluation of the consequences will be 6 months.

### Follow up and data collection

After the workshops, the couples will be followed for 4 months. During this time, they will be asked to read the “My Soul Partner” book carefully with their spouse and complete the exercises and talk about the results together. Also short messages in the field of skill training for sexual relationship will be sent to them by cell phone. Short messages will be based on the content of the book and workshops, which will be sent to recall and review the submitted content. These messages will be transmitted through the creation of a telegram group, which all couples in the intervention group will be members of, and sent as a message every week. Couples will also be able to ask questions about the content of the book and workshops, from the administrators of the group who will be a member of the research team and will provide an answer within 48 h. Data will be collected at the baseline and at four months after the intervention. The original researcher will contact the participants at baseline and at 4 month after the intervention and will coordinate with them to send the questionnaire link. Participants will be asked to respond and submit their responses within a week. If they do not reply within a week, they will be contacted three times. In case of non-response after three times, they will be excluded from the study.

### Database

Participants’ responses to the questionnaire will be visible only to the primary researcher after being submitted online. This data will be saved securely on a server with a strong back up system.

### Sample size

According to the results of studies conducted in Iran about the effect of sexual health education on sexual satisfaction of couples [14], the maximum significant difference was found in sexual satisfaction score between intervention and control groups was 5.38. A sufficient sample size to detect the maximum difference between the intervention and control groups in the sexual satisfaction score with 80% of the power at significant level α = 0, 05 and considering the rate of loss 5% will be 200 people. Thus, 50 couples (*n* = 100) will be assigned to the intervention group and 50 couples (n = 100) will be assigned to the control group.

### Statistical analysis

First the data will be tested for normality, outliers and missing data. Descriptive statistical analysis of the demographic characteristics of two groups will be done to ensure comparability of data in intervention and control groups. To compare the primary (mean score of couples’ sexual information, motivation, behavioral skills and behavior) and secondary (mean score of couples’ sexual satisfaction) outcomes of the study, before and 4 months after the intervention in each group, the paired t-test will be used. Also, independent t test and Analysis of Covariance (ANCOVA) will be performed to compare the outcomes before intervention in two groups and after intervention in two groups. In order to determine the score of sexual satisfaction of couples (female and male score), the principal component analysis will be used and the male and female scores will be converted to one score.

### Ethical considerations

The study was approved by the Ethics Committee of Hamadan University of Medical Sciences with Proprietary ID: IR.UMSHA.REC.1395.435 and agreed by Isfahan University of Medical Sciences (administrators at the research sites). For couples who voluntarily apply for study,the objective and process of the study will be described in detail and fully before entering the study, and the couples can decide whether to enter the study with this information or not. In the informed written consent form that the volunteers must carefully study and sign on before entering the study, it will be stipulated that the content of the study and questions is about your private matters that might be matter of concern. They will be assured that their personal information will not be disclosed at all, their statements will remain confidential and only the research team will have access to it. The actual name of the participants will not be requested at any stage of the study. Participants will be assigned a code that will be identified by the code until the end of the study. It is also emphasized that entry into the study is completely voluntary and participants who are uncomfortable with the sensitivity of the content or questions can be excluded at any stage of the study without any conditions. Due to the sensitivity of the questions, data collection will be done online, and unnamed answer sheet of participants will only be visible to the main investigator so that the participants do not feel uneasy about sharing their answers with other members of the health center that they are referring to. The time of workshops will be determined by the participants and will be considered an appropriate place for holding workshops to provide easy access to all participants. The contents of the book and workshops and messages have been based on the couple’s statements in the qualitative stages and the results of cross-sectional stage, in order to be completely in line with the needs and indigenous religious and cultural values and norms of spouses and do not conflict with them. Only research team members will have access to collected data. The data file will be stored on a secure server with a password, which will be provided only to the research team members.

## Discussion

One of the factors that affects couple’s sexual satisfaction is their familiarity with sexual issue [13]. Sex education includes learning about sexuality, sex, relationships, emotions, creating positive beliefs, values and attitudes [25], which helps couples to have more rational and responsible sexual relationship and prevent sexual dysfunction [14]. In spite of the relatively high level of literacy in Iran that paves the way for educational programs, the existence of a highly effective and efficient primary health care system [26], family planning and national programs for reproductive health of youth [27], the issue of sexual skills training is not adequately addressed. The conservative cultural and religious beliefs of society have led to a lack of valid educational materials and the shift of young people to satellite and pornographic products to meet their sexual needs [28]. The results of the qualitative phase of the present study showed that the participating couples did not have enough information on sexual issues. Many interventions have been designed to promote sexual health of young people in the world that few of them have been successful [29]. Some scholars view the failure of these interventions, the lack of attention to causal studies and their planning without considering psychosocial models as a specific framework [30]. In this study, qualitative study was carried out to identify the factors affecting sexual satisfaction of couples. Accordingly, the IMB model was selected for intervention planning. This model has been used in many sexual health interventions and often used to reduce risky sexual behaviors lead to HIV/AIDS [31–33]. One of the innovations of current study is to use the IMB model to promote healthy sexual behaviors of couples. Interventions are planned based on the results of qualitative and quantitative phase of study tailored to the values and norms governing the sexual life of Iranian couples. Interactive methods will be used to provide educational interventions that will lead to more engaging couples in the subject. Recently, interest has been given to trials that can be done completely or partially over the Internet [34]. The Internet live stats statistical site is estimated the number of Internet users in Iran in 2016 at 39 million [35]. According to the Ministry of Communications, more than 92% of Iranians have mobile phones, and 65% of them are members of one of the social networks, with more than 40 million telegram users in Iran. Therefore, in this study, social networks will be used for recruitment of participants, the measurement of the outcomes of study and the sending educational messages in the follow-up period. Online recruitment of participants may cause unrepresentative sampling; on the other hand, all eligible couples do not go to health centers. Therefore, both call methods through health centers and online call on social networks will be used for sampling so that the maximum numbers of eligible couples have the chance to enter the study. Using the online method to measure the outcomes of the study, while enhancing the confidentiality of the participants, can lead to a decline due to the lack of response from some of the participants. In these cases, it is anticipated that three times a phone call will be conducted by members of the research team and engagement of the couples. In this trial, the impact of the sexual relationship enrichment package on sexual satisfaction of couples will be examined. If the trial is effective, its results will be presented to policy makers for implementation at national level.

## Data Availability

The data that support the findings of this study are available from Fahimeh Bagheri but restrictions apply to the availability of these data, which were used under license for the current study, and so are not publicly available. Data are however available from the authors upon reasonable request and with permission of Ethics Committee of Hamadan University of Medical Sciences.

## Abbreviations

IMB: Information, motivation and behavioral skills model
IMO: In My Opinion
SIMBS: Sexual information, motivation and behavioral skills scale
